# Uptake and predictors of contraceptive use in Afghan women

**DOI:** 10.1186/s12905-015-0173-6

**Published:** 2015-02-13

**Authors:** Mohammad H Rasooly, Mohamed M Ali, Nick JW Brown, Bashir Noormal

**Affiliations:** Afghanistan National Public Health Institute, Ministry of Public Health, Kabul, Afghanistan; Eastern Mediterranean Regional Office, World Health Organization, Cairo, Egypt; Salisbury District Hospital, Salisbury, Wiltshire UK; Aga Khan University, Karachi, Pakistan

**Keywords:** Uptake, Predictors, Contraceptive, Afghan, Women

## Abstract

**Background:**

Afghanistan has one of the world’s highest fertility rates and, related to this, an infant mortality rate far higher than its South Asian neighbors. Contraception enhances family spacing, improves women’s safety in child birth and, as a result, reduces infant and child mortality. Until recently, there has been a paucity of information on the comparative rates of contraceptive practices in the country and socioeconomic correlates of uptake. We aimed to elucidate the factors influencing the use of contraception in Afghanistan using recent, robust national data.

**Methods:**

Using Afghanistan Mortality Survey (AMS) 2010 data, the distribution of Contraceptive Prevalence Rate (CPR) and correlates of contraceptive use among currently married women aged 15–49 years were explored. We initially summarised descriptive data on 25,743 married women and then derived predictors of the use of any form of contraception using a multiple logistic regression model.

**Results:**

The prevalence of self-reported current use of any contraceptive method was 21.8% (95% CI: 20.4-23.4) at the national level though there was a wide variation in practice between provinces. Herat province in the West region had a highest contraceptive prevalence rate of 49.4% while Paktika in the Southeast region had the lowest CPR of 2%. Multiple logistic regression analysis showed that a family size of greater than 6 living children strongly predicted contraceptive use (AOR 7.4 (95% CI:6.1-9.0)). Other independent predictors included: secondary or high level of education (AOR 2.1 (95% CI: 1.8-2.5)) and being in the wealthiest stratum (OR 2.1 (95% CI 1.5-3.0)). Rural residence predicted a lower use of contraception (AOR, 0.72; 95% CI: 0.56-0.92).

**Conclusion:**

Contraceptive uptake rate was low overall with wide inter provincial variation. Strengthening female education, targeting married women in rural area and women with no education may enhance the effectiveness of National Family planning program in Afghanistan.

## Background

Afghanistan is a landlocked country in south Central Asia. Administratively, the country is divided into eight geographical regions, 34 provinces and 398 administrative districts. The 2014/15 estimated population of the country is 26.5 million [1]. Most recent figures from 2010, though better than the previous (2003) data, estimate a total fertility rate of 5.1 child per women, a maternal mortality ratio of 327 per 100,000 live birth, an under-five mortality of 97 and infant mortality of 77 per 1000 live births all amongst the highest in the world. Only 26% of women age 15–49 ever attended school [2,3].

Three decades of internecine war in Afghanistan have damaged the infrastructure, economy and social services. Women’s access to health services has also, as a result, suffered and most women deliver without the presence of a skilled birth attendant [4,5]. There is also evidence to show that the continued high levels of fertility in Afghanistan accompanied with decline in maternal and child mortality may contribute to rapid growth in population, poverty and social issues [6,7]. This is likely to hinder the development of the country already struggling to recover its socioeconomic status. Previous literature suggests that higher education is associated with greater use of contraception but this has not been assessed in detail in Afghanistan.

In response to the sparse national data and inform future policy, the Afghanistan Mortality Survey (AMS) was conducted in 2010. Its aim was to provide information on the levels, trends, differentials, and causes of mortality and levels and differentials in related health and health care indicators. Recognising the potential health benefits of family planning, the AMS additionally collected data on fertility, marriage and family planning [2]. The National Family Planning Program was established in 2002 under the Reproductive Health Directorate of the Ministry of Public Health. Since then, family planning related activities have been integrated into a primary health package called Basic Package of Health Service (BPHS) and secondary and tertiary health package called the Essential Package of Hospital Services (EPHS) through more than 2000 public health facilities. In addition, family planning methods are also available through number of charities such as Marie Stopes International, the Afghan Family Guidance Association, Future Group and pharmacies.

Currently, oral contraceptive pills, the intra-uterine device (IUD), male condom and injectable contraceptives are widely available throughout the country. In some cities female sterilization and implant are also accessible. We sought to test this and other socioeconomic relationships using routinely collected and robust data. With this in mind, our study set out to provide a picture on distribution and correlation of contraceptive use in Afghanistan utilizing AMS 2010 data. The dataset is publically available at measure DHS website [8].

## Methods

The Afghanistan Mortality Survey 2010 used the sampling frame prepared for Population and Housing Census (PHC) by Central Statistics Organization (CSO). It used a two-stage cluster sampling design to generate a nationally representative sample of households. In the first stage, the clusters were selected from the sampling frame. Households were then selected from each cluster allowing for stratification of urban and rural areas. The remoteness index is constructed using a similar methodology as the wealth index to measure the lack of infrastructure and/or distance services centers and it is constructed at the cluster level instead of the household level. The information is captured through a series of questions to the village leader or a knowledgeable person. For security reasons, the rural areas of Kandahar, Helmand, and Zabul provinces of South zone were excluded from the survey. Further details of the method used by region can be found in the main report of AMS 2010 [2].

Information for the household was obtained through personal interviews with an adult household member usually the head of the household. In the vast majority (96.7% cases) men, husband or father in law, were the head of households with the household questionnaire. The questionnaires were based on the validated Demographic and Health Surveys Core Questionnaires, modified for use in Afghanistan. All staff were intensively trained for three weeks through both classroom lectures and field practice. Female surveyors interviewed all women 12–49 years of age within the selected households using the women’s questionnaire. Maintaining the privacy and confidentiality during the interview were emphasized throughout the surveyors’ training and field monitoring. Efforts were made to interview women in privacy uninfluenced by their husband or mother in law. Since the interviews were conducted by a female interviewer known to the community, respondents were able to disclose freely relevant information about the use of any contraceptive methods by themselves or their husbands.

The interview sought information on a woman’s background (age, education, ethnicity, and marital status), their use of health services, on each of their children and the survival status of each child. The current use of contraceptive was assessed by the question “Are you currently doing something or using any method to delay or avoid getting pregnant? Where an affirmative was given, a further question” which method are you using?” was asked. Modern methods were defined as the oral contraceptive pill, IUD, injectable, condom, emergency contraception, implants and male and female sterilization. Traditional methods were as follows: periodic abstinence (rhythm), withdrawal and Lactation Amenorrhea Method (LAM).

### Data analysis

The primary outcome measure was current use of any contraceptive method, a dichotomous variable, in non-pregnant married women. In the descriptive analysis, we illustrated the distribution of current contraceptive use by national, regions and provinces. Due to small numbers, data from Urozagan, Nimroz and Punisher (like the insecure regions) were not analysed further. To ensure representativeness across the country, data used were weighted according to survey sample. We then conducted exploratory analyses of the relationships between selected demographic and socioeconomic variables and current contraceptive use using current use of any contraception as a dichotmised outcome. Categorical and ordinal variables (place of residence, region, level of education, and experience of child death) were used as coded in the survey questionnaire, the two latent variables (wealth quintiles and remoteness) were computed from a set of variables collected in the household questionnaire using factor analysis and incorporated in the final survey dataset as categorical variables with five groups. In our analysis, however, we have grouped the three continuous variables age, age at marriage and number of living children using standard categories presented in most DHS surveys to ensure that there are enough numbers in each category for robust analysis. Bivariate outcomes and exposures were assessed by Chi squared test and potential predictors were assessed in a multiple logistic regression model using backward elimination method. The results of logistic regression analysis are given as adjusted odds ratios, with 95% confidence interval and *p* values. All analysis was conducted in STATA version 13 taking into account the survey sample and cluster design and weights.

## Results

### Descriptive analysis

Members of 22,351 households were assessed for criteria for interview.

The analyses reported here are based on information on current use of contraceptive methods provided non-pregnant women aged between 15 and 49 years who numbered 25,743. Use of any method of family planning was estimated as 22% with breakdown as follows (Table 1): injectable contraceptives (6.5%), oral contraceptive pills (5.3%); LAM (3.6%); male condoms (1.7%); the intrauterine contraceptive device (1.3%); traditional methods (1.9%) and female sterilization (1.4%).

**Table 1:** **Proportion of currently married women age 15–49 used contraceptive (%) by province and region**

	**Number of married women age 15–49 interviewed**	**Percent of currently using contraceptive**	**95%** **CI**
**Provinces**			
Kabul	2,678	35.1	32.0-38.3
Kapisa	462	36.6	28.2-45.9
Parwan	615	40.9	35.7-46.3
Wardak	540	12.5	7.3-20.5
Logar	378	34.2	27.2-42.0
Nangarhar	2,369	21.4	18.8-24.0
Laghman	601	18.1	12.6-25.0
Baghlan	815	15.0	11.8-19.0
Bamyan	270	25.5	12.4-45
Ghazni	1,044	13.4	8.8-20
Paktika	667	2.1	0.6-7.0
Paktya	962	8.4	6.1-11.0
Khost	760	17.8	14.6-22.0
Kunar	1,171	6.2	4.1-9.0
Nuristan	356	5.7	2.7-12.0
Badakhshan	1,102	24.9	17.2-34.5
Takhar	1,001	6.9	4.9-10.0
Kunduz	904	10.2	7.8-13.0
Samangan	385	14.0	8.9-21.0
Balkh	1,217	23.0	17.9-29.0
Sari Pul	559	15.4	12.5-19.0
Ghor	574	11.3	7.3-17.0
Daykundi	645	39.8	31.0-49.0
Jawzjan	639	15.3	10.5-22.0
Faryab	1,162	6.7	4.5-10.0
Badghis	526	8.4	4.6-15.0
Herat	1,638	49.4	44.2-54.5
Farah	666	31.1	21.5-43
**Regions**			
North Eastern	3,823	14.6	11.8-18.0
Northern	3,960	15.0	12.8-17.6
Western	3,405	33.0	28.0-38.0
Central Highlands	915	35.6	26.9-45
Capital	4,791	33.6	30.9-36.0
Eastern	4,497	15.8	13.4-18.0
Southern	1,963	24.1	19.3-29.6
South Eastern	2,388	9.6	7.4-12.0
**Total**	**25,743**	**21.8**	**(20.4-23.4)**

**Figure 1:**
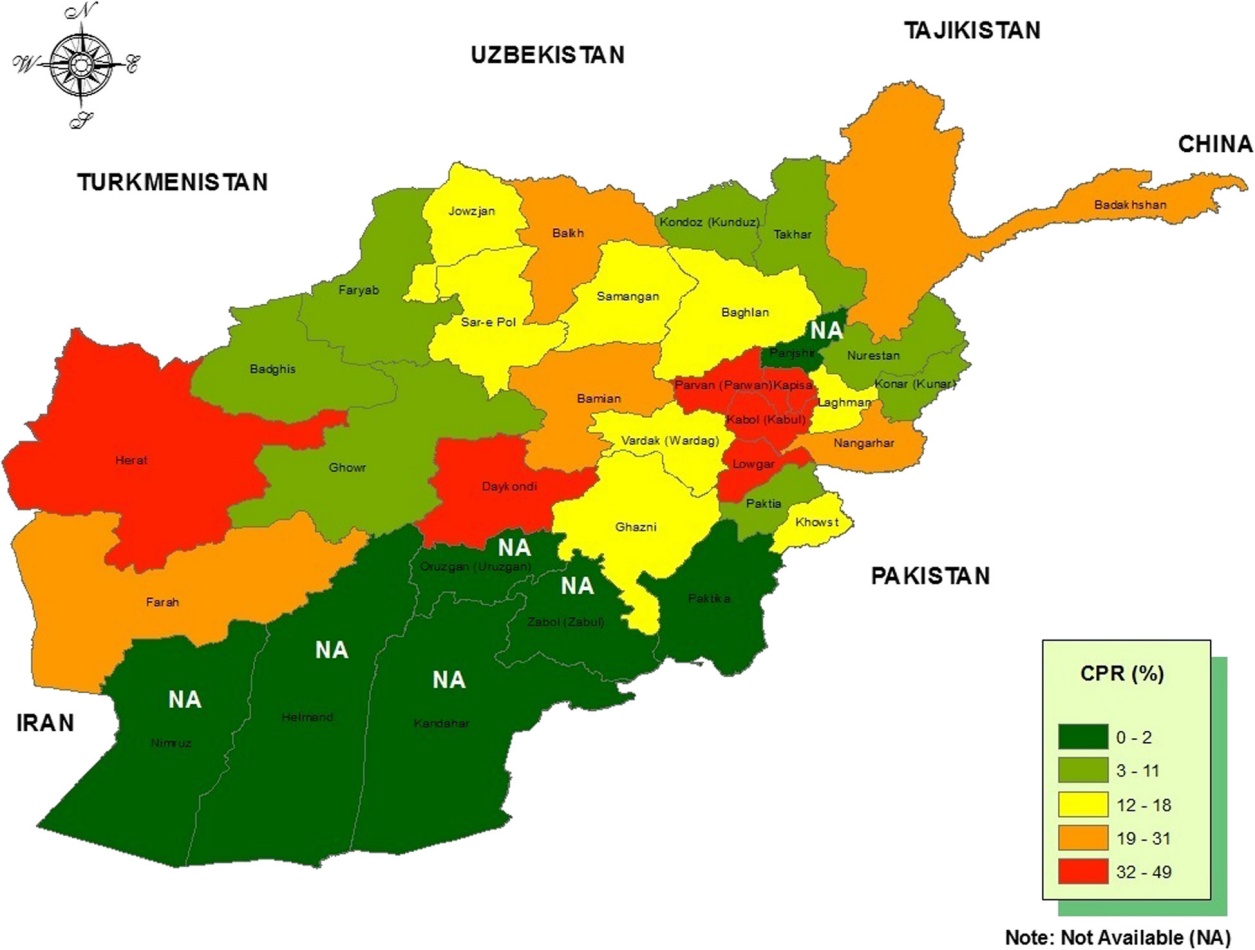
**Distribution of contraceptive prevalence rate by provinces (%).**

Table 1 shows the percent distribution of currently married women age 15–49 who were using specific family planning methods at the time of the survey, by province and regions. Herat province had the highest contraceptive prevalence rate (about 49%), while Paktika had the lowest contraceptive prevalence rate of 2% (Figure 1). At the regional level, Central Highland, Capital and Western regions, have the largest percentage of currently married women currently using contraception, while the Southeast region had the smallest percentage.

Use of contraceptive varied by age group, the lowest use being in the youngest age group (14%). Uptake increased successively by age group to 26.6% in the 35–49 year group. There were significant differences in the current use of contraceptive methods among subgroups of currently married women. The use of family planning among urban women was double that of rural women (36 percent and 18 percent, respectively). Contraceptive use was more than twice as high in the Central Highlands as in the North, East and Northeast regions. There was a significant association between use of contraceptive and level of education with and increase from 20 percent among women with no formal education to 33 percent among women with primary education to 39 percent among women with secondary and higher education (p < 0.001).

Wealth also was associated with the women’s contraceptive use: only 17% of married women in the lowest wealth quintile were users compared to 33.9% in the highest wealth quintile (Table 2).

**Table 2:** **Current use of contraceptive by socio-demographic characteristics among married women 15–49 (Bivariate analysis)**

		**n**	**Contraceptive prevalence rate**	**95%** **CI**	**P. Value**
**Residence**					
	Urban	4,934	36.4	33.9-39.0	<0.001
	Rural	20,809	18.4	16.8-20.0	
**Region**					
	North Eastern	3,823	14.6	11.8-18.0	<0.001
	Northern	3,960	15.0	12.8-17.6	
	Western	3,405	33.0	28.0-38.0	
	Central Highland	915	35.6	26.9-45.0	
	Capital	4,791	33.6	30.9-36.0	
	Eastern	4,497	15.8	13.4-18.0	
	Southern	1,963	24.1	19.3-29.6	
	South Eastern	2,388	9.6	7.4-12.0	
**Education**					
	No education/Madrasa	22,914	20.1	18.6-21.7	<0.001
	Primary	1,485	33.0	29.9-36.0	
	Secondary/Higher	1,344	39.1	35.7-42.6	
**Age group**					
	15-24	7,221	14.0	12.6-15.6	<0.001
	25-34	9,220	23.2	21.3-25.0	
	35-49	9,302	26.6	24.7-28.6	
**Age at first marriage**					
	Less than 16	9,035	24.7	22.7-26.9	<0.001
	16-19	8,892	21.2	19.5-23.0	
	19+	7,816	19.3	17.8-20.9	
**Number of living children**					
	0-1	5,800	7.4	6.5-8.5	<0.001
	2-3	6,434	21.0	19.2-22.9	
	4-5	6,033	27.3	25.0-29.6	
	6+	7,476	29.4	27.2-31.7	
**Child death experiences**					
	No	20,005	20.9	19.4-22.5	<0.001
	Yes	5,738	25.1	23.1-27.0	
**Remoteness**					
	Most remote	6,621	18.4	15.9-21.0	0.0019
	2	7,255	22.9	19.8-26.0	
	3	5,110	21.3	18.4-24.5	
	4	4,189	21.6	18.0-25.7	
	Least remote	2,568	29.3	25.2-33.8	
**Wealth quintiles**					
	Poorest	5,115	17.0	14.1-20.5	<0.001
	Poorer	5,328	19.8	17.5-22.0	
	Middle	5,057	17.8	15.7-20.0	
	Richer	5,153	20.7	18.6-22.9	
	Richest	5,090	33.9	31.5-36.0	
**Mass media exposure**					
	No exposure	6,864	17.0	14.6-19.6	<0.001
	Radio only	12,301	19.5	17.8-21.0	
	Radio and TV	6,578	31.3	29.1-33.5	

### Multivariate analysis

We found that larger family size strongly predicted contraceptive use. The AOR for women with 6+ living children was estimated as 7.4 (95% CI: 6.1-9.0). Additional independent predictors included: greater age 0.81 (95% CI: 0.69-0.95);wealth index 2.1 (95% CI: 1.50-2.95); place of residence 0.72 (95% CI: 0.56-0.92); and education 2.10 (95% CI: 1.80-2.45). Media exposure, remoteness of the households, age at first marriage were not associated with use of current use of contraception (Table 3).

**Table 3:** **Factors associated with use of contraceptive by socio-demographic characteristics in married women 15–49 (Multivariate analysis)**

		**AOR**	**95% CI**	**p value**
**Residence**				
	Urban	1.00		
	Rural	0.72	0.56-0.92	0.007
**Region**				
	North Eastern	1.00		
	Northern	0.97	0.72-1.31	0.843
	Western	2.90	2.06-4.08	<0.001
	Central Highland	4.07	2.48-6.70	<0.001
	Capital	1.84	1.36-2.48	<0.001
	Eastern	0.89	0.65-1.20	0.440
	Southern	1.42	0.97-2.06	0.068
	South Eastern	0.51	0.35-0.75	0.001
**Education**				
	No education/Madrasa	1.00		
	Primary	1.60	1.36-1.81	<0.001
	Secondary/Higher	2.10	1.80-2.45	<0.001
**Age group**				
	15-24	1.00		
	25-34	0.89	0.8-1.01	0.070
	35-49	0.81	0.69-0.95	0.012
**Number of living children**				
	0-1	1,00		
	2-3	3.72	3.23-4.30	<0.001
	4-5	5.77	4.87-6.84	<0.001
	6+	7.40	6.06-9.02	<0.001
**Wealth quintiles**				
	Poorest	1.00		
	Poorer	1.33	1.06-1.69	0.016
	Middle	1.39	1.05-1.84	0.022
	Richer	1.67	1.22-2.27	0.001
	Richest	2.10	1.50-2.95	<0.001

## Discussion

We found low rates of contraceptive use among married, non-pregnant women in Afghanistan with further reduction in uptake associated with the poverty, low level of education and younger age. Number of years of education might provide married women on the better awareness on advantages of birth spacing and use of contraceptive. Similarly, educated women could have positive health seeking behavior. The increase number of living children as mentioned strongly associated with use of contraceptive among currently married women. The findings from the studies conducted in two cities of Pakistan were also found that number of living children was strongly associated with use of contraceptive methods [9,10].

Though uptake has increased since previous surveys (perhaps a result of the Basic Package of Health Services (BPHS) introduced in 2003), male use has stalled with no increase in the use of the male condom from 2006–2011 [11-13]. This is probably a reflection of the fact that family planning information and services in Afghanistan are not targeted towards men but have been provided in the context of maternal and child health. Although contraceptive use has increased both nationally and in the provinces in recent years, women who were living in rural area are less likely to use any contraceptive methods than their urban counterparts

Despite the proportion of contraceptive use was increasing overall, the reliance on the traditional methods remained constant during last decade. The low rates of sterilisation could be because it is not part of National Family Planning Program in Afghanistan, low availability outside cities and, given the conservative nature of Afghan society, less likely to be acceptable to religious leaders.

Our findings are compatible with those from other studies. A comparative study of contraceptive use in Nigeria revealed similar rural urban disparity in use of contraceptive [14]. In the former Soviet Union, Janevic et al. showed that women from poorest communities less likely to use contraceptive methods than their wealthier counterparts [15] a finding that has also been made in Malawi [16]. Our study discovered that educational level was positively associated with contraceptive use in Afghanistan and women with no or informal education were the least likely to use any contraceptive methods than women with secondary and higher education. Similar patterns were also observed in elsewhere [17-24]. Number of years of education might provide married women on the better awareness on advantages of birth spacing and use of contraceptive. Similarly, educated women could have positive health seeking behavior.

The study had number of strengths. It was based on near complete national coverage and represents by far the most comprehensive sample to date.

There were a number of limitations. As this study was cross-sectional survey, a definite causal relationship between the outcome measure and the exploratory variables could not be established. There may also have been biases resulting from the exclusion of a third of the rural areas in the South and, possibly, despite great sensitivity on the part of the interviewers, a reluctance to divulge information amongst the women. We were also unable to establish qualitative reasons for use and non-use of contraception again perhaps a reflection of cultural sensitivities.

## Conclusion

Rates of contraceptive uptake are low in Afghanistan and rural, uneducated women are less likely to use any of the standard methods. The family planning programme must expand its activities to inform this vulnerable group, involve men and promote longer acting or permanent contraception such as the IUD and female sterilization.

## Abbreviation

AMS: Afghanistan Mortality Survey
AOR: Adjusted odds ratios
BPHS: Basic package of health services
CPR: Contraceptive prevalence rate
CSO: Central Statistics Organization
DHS: Demographic and Health Survey
EAs: Enumerated areas
PHC: Population and Housing Census
PSU: Primary sampling unit
